# A Herpesviral Lytic Protein Regulates the Structure of Latent Viral Chromatin

**DOI:** 10.1128/mBio.00633-16

**Published:** 2016-05-17

**Authors:** Priya Raja, Jennifer S. Lee, Dongli Pan, Jean M. Pesola, Donald M. Coen, David M. Knipe

**Affiliations:** a Department of Microbiology and Immunobiology, Harvard Medical School, Boston, Massachusetts, USA; b Program in Virology, Harvard Medical School, Boston, Massachusetts, USA; c Department of Biological Chemistry and Molecular Pharmacology, Harvard Medical School, Boston, Massachusetts, USA

## Abstract

Latent infections by viruses usually involve minimizing viral protein expression so that the host immune system cannot recognize the infected cell through the viral peptides presented on its cell surface. Herpes simplex virus (HSV), for example, is thought to express noncoding RNAs such as latency-associated transcripts (LATs) and microRNAs (miRNAs) as the only abundant viral gene products during latent infection. Here we describe analysis of HSV-1 mutant viruses, providing strong genetic evidence that HSV-infected cell protein 0 (ICP0) is expressed during establishment and/or maintenance of latent infection in murine sensory neurons *in vivo*. Studies of an *ICP0* nonsense mutant virus showed that ICP0 promotes heterochromatin and latent and lytic transcription, arguing that ICP0 is expressed and functional. We propose that ICP0 promotes transcription of LATs during establishment or maintenance of HSV latent infection, much as it promotes lytic gene transcription. This report introduces the new concept that a lytic viral protein can be expressed during latent infection and can serve dual roles to regulate viral chromatin to optimize latent infection in addition to its role in epigenetic regulation during lytic infection. An additional implication of the results is that ICP0 might serve as a target for an antiviral therapeutic acting on lytic and latent infections.

## Importance

Latent infection by viruses usually involves minimizing viral protein synthesis so that the host immune system cannot recognize the infected cells and eliminate them. Herpes simplex virus has been thought to express only noncoding RNAs as abundant gene products during latency. In this study, we found genetic evidence that an HSV lytic protein is functional during latent infection, and this protein may provide a new target for antivirals that target both lytic and latent infections.

## INTRODUCTION

The herpesviruses are characterized by the ability to establish a latent infection, in which no infectious virus is found, following an acute infection in the host organism (1). Herpes simplex virus (HSV) initially productively infects epithelial tissue (“lytic” infection), from which it spreads to establish a latent infection in sensory neurons (2). HSV directly causes significant morbidity and mortality; moreover, it promotes HIV infection (2). The existing antiherpesvirus drugs target lytic infection; thus, therapeutics to cure latent infections will require new antiviral strategies. Additional knowledge about the mechanisms of HSV latent infection is needed to design therapeutics that target latent infection.

HSV lytic and latent infections have been thought to occur along distinct pathways and to be driven by different viral gene products (2, 3). The HSV DNA genome is packaged in virions without associated histones, but during lytic infection, the HSV genome enters the host cell nucleus and is rapidly associated with heterochromatin, which is then modified to euchromatin (4–6). Virion protein 16 (VP16) assembles into a complex with host proteins, and the complex recruits chromatin-remodeling and -modifying proteins to viral immediate-early (IE) promoters to reduce total histone and heterochromatin occupancy and to increase euchromatin marks on IE genes (7). One of the resulting IE proteins, infected cell protein 0 (ICP0), reduces total histone loading and heterochromatin and increases euchromatin on the rest of the genome (5, 6). Known mechanisms of ICP0 action in promoting gene expression include inactivation of repressive effects of PML/Sp100/Daxx/ATRX (8–11), disruption of the CoREST-histone deacetylase (HDAC) complex (12), recruitment of the CLOCK histone acetyltransferase (13), and degradation of IFI16 (14).

In contrast to lytic infection, where the HSV genome is rapidly heterochromatinized within 1 to 2 h of infection, the HSV-1 latent genome accumulates histones gradually over days and weeks following infection (15, 16). Previous experiments in the mouse ocular infection model indicate that histone H3 association with viral genomes in trigeminal ganglia (TG) takes up to 7 days postinfection (dpi), whereas H3K27me3 association was not detectable until 10 to 14 dpi (15).

During latent infection of mice, HSV-1 does not produce infectious virus, and although many viral genes are expressed, their expression levels are rather low, with the only abundant viral transcripts accumulating from the latency-associated transcript (LAT) region (17–23). The HSV latent genome is organized in ordered nucleosomes (24), and the silencing of viral genes during latent infection correlates with the accumulation of histones and heterochromatin modifications during the establishment of latency (16). Viral lytic promoters are associated with chromatin characterized by constitutive H3K9me3 and facultative H3K27me3 heterochromatin modifications, while the *LAT* promoter is associated with bivalent chromatin characterized by both euchromatin and heterochromatin modifications, without any role for DNA methylation (15, 16, 25–27). This has led to the hypothesis that an epigenetic switch initiates the transition of the viral genome from lytic to latent infection.

The LATs play several potential roles in promoting latent infection in that they have been implicated in promoting cell survival by inhibiting apoptosis or other mechanisms of cell death (28, 29), repressing lytic gene expression (30, 31), and facilitating heterochromatin accumulation on the viral genome (16, 25). Enrichment of H3K27me3 is promoted by LATs in our system and corresponds to a significant accumulation of the PRC2 component Suz12 on lytic promoters by 14 dpi but not the PRC1 component Bmi1 (15, 25, 26). Interestingly, recruitment of Suz12 occurs independently of LAT transcription, suggesting that additional viral mechanisms promote H3K27me3 accumulation (15). Whereas heterochromatin modifications were previously believed to represent a terminally silenced genome, it has become increasingly clear that latent infection is not characterized by permanent and static repression of the viral genome but represents an ongoing dynamic balance mediated by both the virus and the infected cell (17, 18, 32, 33).

The lytic and latent infection pathways have been thought to be distinct and involve separate viral gene products. Several important functions have been described for the IE ICP0 protein during lytic infection. ICP0 promotes viral lytic replication at low multiplicities of infection (MOIs) and to different extents in different cell types (34). This involves ICP0 promoting reversal of host cell epigenetic silencing of the viral genome (6) and inhibition of host restriction mechanisms, as described above. ICP0 has been shown to promote viral reactivation (35–38), but this was thought to be due to its functions in promoting lytic infection.

Interestingly, there is evidence that, in latent infection, low levels of *ICP0* and other lytic transcripts are detectable in the absence of reactivation (17, 20, 39–42). Furthermore, there is evidence that the *ICP0* gene promoter is active at some point during the establishment of latency in at least one-third of latently infected neurons (41). Also, recently, a mutation that results in increased expression of ICP0 in neurons was shown to lead to increased expression of lytic genes in ganglia during establishment and maintenance of latency (33).

One study involving infection of sensory neurons *in vitro* resulted in a report that ICP0 is necessary for efficient establishment of latent infection (43). However, those were *in vitro* experiments involving incubation of neurons with the antiviral drug acyclovir during infection. We therefore examined the potential role of ICP0 in latent infection *in vivo* in a mouse infection model. In contrast to lytic infection results, we found that ICP0 promoted both *LAT* and lytic gene expression in latently infected ganglia as well as total histone and heterochromatin loading on the latent HSV genome. This new function of ICP0 raises the potential of antivirals targeting it for use as therapeutics for HSV latent infection.

## RESULTS

### ICP0-null mutant viruses show reduced replication in mouse ocular infection.

**FIG 1:**
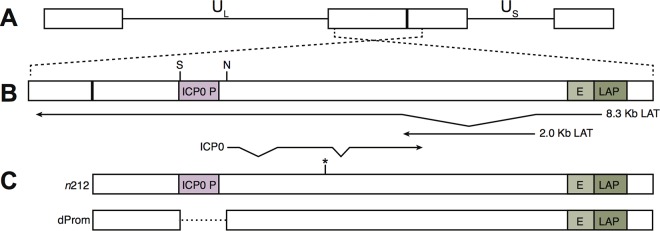
HSV-1 genomic map of the *ICP0* region. (A) Shown here are the unique short region (U_S_) and unique long region (U_L_) of HSV-1 flanked by inverted repeats. (B) Magnified view of the *ICP0* region, which encodes ICP0 transcribed from its promoter (ICP0 P), the antisense primary 8.3-kb *LAT* transcript transcribed from the LAT promoter (LAP), and the stable 2-kb *LAT* intron. E, *LAT* enhancer. (C) The *d*Prom mutant virus has a 711-bp deletion between the NcoI (N) and StuI (S) sites depicted on this map. The *n*212 mutant virus contains a nonsense mutation in codon 212 in exon 2 of the *ICP0* transcript (indicated by an asterisk).

**FIG 2:**
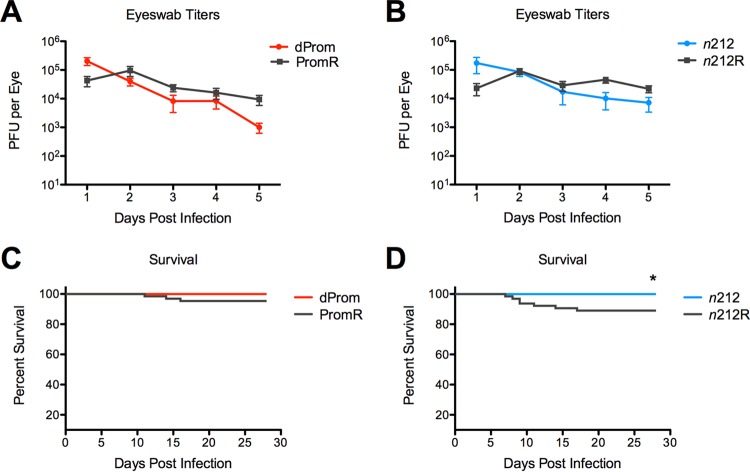
Acute replication in mouse corneal epithelia and survival of mice infected with the ICP0^−^ and ICP0^+^ viruses. Mice were infected with 2 × 10^6^ PFU/eye of *ICP0* mutant *d*Prom virus (A and C) and *n*212 virus (B and D) and 2 × 10^4^ PFU/eye of rescued PromR virus (A and C) and *n*212R virus (B and D). (A and B) Viral replication in mouse corneal epithelia. Eye-swabbing procedures were carried out from 1–5 dpi, and viral titers were determined. (C and D) Mouse survival over 28 dpi was compared for mice that were infected with ICP0-mutant and ICP0-rescued viruses in three independent experiments, and survival curves are presented. Statistical significance was evaluated using the log rank Mantel-Cox test (*P* < 0.001).

We wished to test the role of the HSV-1 ICP0 protein in latent infection, and previous studies had utilized an HSV-1 mutant virus (7134) with a *lacZ* insertion in the *ICP0* gene (44). This virus shows altered *LAT* expression (36; our unpublished results), which complicates interpretation of the results. Other studies had compared the HSV-1 *n*212 ICP0 nonsense mutant virus to wild-type (WT) virus but did not analyze a rescued virus (38) (Fig. 1). The *n*212 mutant virus has minimal changes in the *LAT* coding sequences; therefore, we utilized the HSV-1 *n*212 ICP0 nonsense mutant and constructed a rescued *n*212R virus. We also constructed the HSV-1 *d*Prom *ICP0* promoter deletion mutant virus and generated the PromR rescued virus, as described previously (6). The *n*212R and PromR rescued viruses were shown previously to have replication kinetics and gene expression and chromatin profiles similar to those of WT KOS virus in acute infection (6). Previous studies had shown that ICP0-negative (ICP0^−^) viruses show reduced ocular replication, so higher doses of the mutant viruses were needed to establish comparable levels of latent infection (38, 45). To determine the relative doses of ICP0^+^ and ICP0^−^ viruses that gave equivalent titers in the eye at peak times of replication (2 to 3 dpi), we infected mice with various doses of the viruses by the corneal route, collected eye swabs daily up to 5 dpi, and titrated the virus in the tear fluid on U2OS cells, which complement the growth of ICP0 mutant viruses (46), to assess acute replication in the eye. We observed viral titers at 2 dpi in mice infected with 2 × 10^6^ PFU/eye of the *ICP0* mutant virus *d*Prom or *n*212 that were equivalent to those seen with mice infected with 2 × 10^4^ PFU/eye of the PromR or *n*212R rescued viruses, but then the ICP0*^+^* virus titers increased to levels above the ICP0^−^ virus titers, with statistically significant differences by 4 to 5 dpi (*P* < 0.05) (Fig. 2A and B). All of the ICP0^−^ virus-infected animals survived, but reduced numbers of ICP0^+^ virus-infected animals survived, which was significant for the comparison of *n*212 results to *n*212R results (*P* < 0.001) (Fig. 2C and D). These results were consistent with increased replication of ICP0^+^ viruses in ganglia, increased lethality, and increased spread of virus from the ganglia back to the cornea. These inocula were used for all subsequent experiments because they gave equivalent peak viral titers in the eye.

### Total chromatin and heterochromatin profiles on lytic and latent gene sequences in genomes of ICP0^+^ and ICP0^−^ viruses.

**FIG 3:**
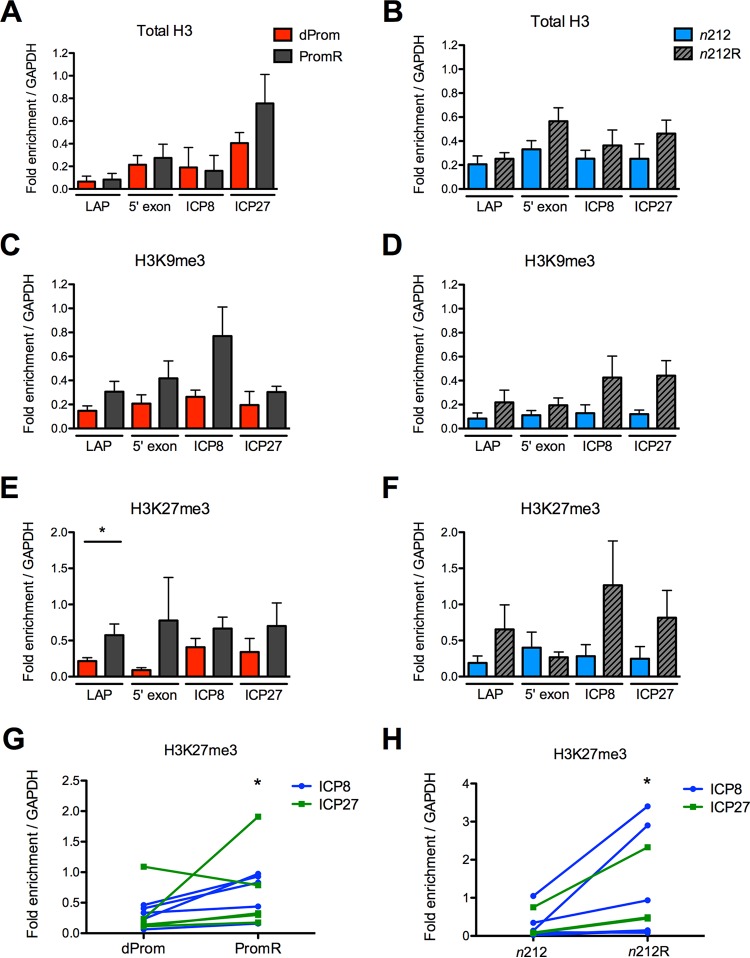
Chromatin profile of latent ICP0-expressing and ICP0-mutated genomes. Chromatin from latently infected trigeminal ganglia was evaluated by ChIP for total H3 (A and B), H3K9me3 (C and D), and H3K27me3 (E to H). ChIP results are presented for viral regions *LAP*, the *LAT* 5′ exon, *ICP8*, and *ICP27* normalized to the cellular GAPDH gene. Data from the *ICP0* promoter deletion virus *d*Prom and the cognate WT rescued virus, PromR, are compared in panels A, C, E, and G. Data from the *ICP0* nonsense mutant *n*212 and its rescued virus *n*212R are compared in panels B, D, F, and H. H3K27me3 association with lytic genes ICP8 and ICP27 is shown in panels G and H. Note that each pair of data points is derived from individual ChIP experiments performed in parallel. Six independent ChIP experiments were performed on samples obtained from three independent infections of 20 to 30 mice per group. Statistical significance was evaluated using the Wilcoxon matched-pair signed-rank test (*P* < 0.05).

To measure the effects of ICP0 on chromatin association with latent viral genomes, we evaluated the total chromatin and heterochromatin profiles on HSV-1 latent genomes from ICP0^−^ and ICP0^+^ viruses during latent infection. Mice were sacrificed at 28 dpi, and trigeminal ganglia were harvested. Chromatin immunoprecipitation (ChIP) analysis was performed to measure the association of histone H3, H3K9me3, and H3K27me3 with the viral *ICP8* promoter, *ICP27* promoter, or *LAT* promoter (*LAP*) or the *LAT* enhancer (E; 5′ exon) relative to cellular GAPDH (glyceraldehyde-3-phosphate dehydrogenase) by quantitative PCR (qPCR) (Fig. 3).

We found approximately equal amounts of viral DNA in chromatin preparations recovered from ganglia infected with mutant viruses and those infected with rescued viruses. We also found that, in general, all the sequences assayed on the latent viral genomes were associated with heterochromatin markers of silencing such as H3K27me3 and H3K9me3, as observed previously (16, 25). However, in contrast to the effect of ICP0 on chromatin during lytic infection (6), we found increased levels of total histones and heterochromatin on the ICP0^+^ viruses relative to the ICP0^−^ viruses (Fig. 3, panels A to F). In particular, there was a significant increase in H3K27me3 levels on the *LAP* in the PromR genome (*P* < 0.05) (Fig. 3). When the H3K27me3 values for the lytic gene promoters were grouped for ICP0^+^ and ICP0^−^ viruses, we observed a statistically significant increase in H3K27me3 modifications on the PromR and *n*212R viruses relative to the *d*Prom and *n*212 viruses, respectively (Fig. 3, panels G and H).

The trends of H3K9me3 modification were similar to those seen with the H3K27me3 modification but were not statistically significant. The two ICP0^−^ mutant virus strains, *n*212 and *d*Prom, showed similar phenotypes, arguing that ICP0 promotes increased heterochromatin modifications, in particular, the H3K27me3 modification, at lytic promoters and on the *LAT* promoter during latent infection.

**FIG 4:**
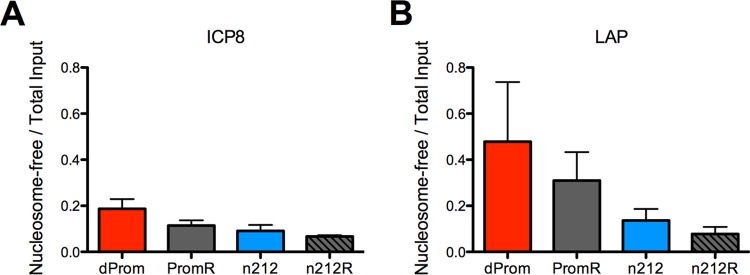
FAIRE profile of latent ICP0-expressing and *ICP0* mutant genomes. Chromatin from latently infected trigeminal ganglia was evaluated by FAIRE to compare the proportion of nucleosome-free viral DNA relative to the total input viral DNA amount in ICP0-expressing (PromR or *n*212R) and ICP0 mutant (*d*Prom or *n*212) viruses. FAIRE results are presented for *ICP8* (A) and *LAP* (B) regions.

To examine the latent viral chromatin using an antibody-independent method of chromatin analysis, we used the formaldehyde-assisted isolation of regulatory elements (FAIRE) method (47) to measure the ratio of nucleosome-free DNA to nucleosome-bound DNA in the ICP0^−^ viruses relative to the ICP0^+^ viruses. This technique uses phenol extraction to recover nucleosome-free DNA in the aqueous phase. We observed increased amounts of nucleosome-free DNA from the ICP0^−^ viruses relative to the ICP0^+^ viruses at both the *ICP8* promoter and *LAP* regions (Fig. 4). Although the differences did not reach statistical significance (*P* < 0.09 for the *ICP8* promoter on *d*Prom compared to PromR), the FAIRE results supported our results from ChIP experiments and argued that, in latency, ICP0-expressing viruses were associated with greater levels of repressed and nucleosome-bound chromatin.

### Viral RNA expression during latent infection with ICP0^−^ and ICP0^+^ viruses.

**FIG 5:**
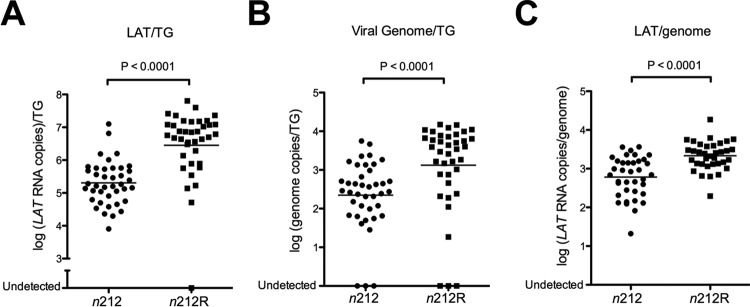
*LAT* expression in the *n212* virus relative to the *n*212R virus. *LAT* transcripts were quantified in latently infected trigeminal ganglia by qRT-PCR. Viral transcripts were measured with primers specific to the *LAT* intron and normalized relative to a cellular control. Viral genomes from the same ganglia were measured by qPCR and normalized to cellular DNA. Statistical significance was evaluated using the Mann-Whitney test. (A) *LAT* transcripts/TG (*P* < 0.0001). (B) Viral genomes in latently infected trigeminal ganglia (*P* < 0.0001). (C) LAT transcripts/genome (*P* < 0.0001).

To determine whether the levels of latent infection transcripts were altered with an ICP0^−^ virus relative to its ICP0^+^ rescued partner, we performed quantitative reverse transcription PCR (qRT-PCR) to quantify *LAT* and lytic transcripts in *n*212 and *n*212R latently infected trigeminal ganglia. Viral transcripts measured with primers specific to the *LAT* intron and to lytic transcripts were normalized to a cellular control. Viral genomes from the same ganglia were measured by qPCR and normalized to cellular DNA. Ganglia from mice infected with *n*212R virus expressed significantly (14-fold) higher levels of the stable *LAT* intron than ganglia from those infected with *n212* virus (*P* < 0.001) (Fig. 5A). The total amount of viral DNA isolated was also significantly higher in ganglia latently infected with the ICP0^+^ virus than in ganglia latently infected with the ICP0^−^ virus (Fig. 5B). Nevertheless, *LAT* expression per viral genome was significantly (3.6-fold) higher in ganglia infected with the ICP0^+^ virus than in ganglia infected with the ICP0^−^ virus at day 28 (Fig. 5C).

We also assayed for lytic viral transcripts from the ICP0, ICP27 (immediate-early), thymidine kinase (tk) (early), and glycoprotein C (gC) (late) genes*.* As expected, especially given the low inoculating dose of the ICP0^+^ virus, the levels of these lytic gene transcripts were very low. Because the transcript levels were low, we initially scored ganglia as positive or negative for detectable transcripts. About one-third of ganglia from mice infected with the *n*212R ICP0^+^ virus showed detectable *ICP0* expression, while only 1/10 of ganglia infected with *n*212 mutant virus did, which was a significantly smaller proportion (Fisher’s exact test; Table 1). Similarly, a larger proportion of ganglia infected with the *n*212R ICP0^+^ virus showed detectable *ICP27*, *tk*, and *gC* gene transcripts, and these differences were significant for the *tk* and *gC* gene transcripts (Table 1). When the ganglia were analyzed for numbers of lytic transcripts per genome, there were significantly higher levels of all four lytic transcripts found in ganglia from mice infected with the *n*212R ICP0^+^ virus than in ganglia from mice infected with the *n*212 mutant virus (Mann-Whitney test; Table 1). In total, these results showed that ICP0 promotes increases in levels of both *LAT* and lytic transcripts.

**TABLE 1:** Expression of viral lytic transcripts during latent infection

Virus (parameter)	Parameter value for indicated gene			
	*ICP0*	*ICP27*	*tk*	*gC*
*n*212 (+/total)^a^	4/40	3/40	3/40	6/40
*n*212R (+/total)	12/37	8/37	10/37	15/37
*P* value^b^	0.023	0.110	0.032	0.020
*n*212 (RNA/genome)^c^	0.0019	0.0014	0.0014	0.0020
*n*212R (RNA/genome)	0.0042	0.0035	0.0026	0.0043
*P* value^d^	0.033	0.045	0.023	0.024

a +/total, number of RNA-positive ganglia/total number of ganglia assayed. Ganglia were scored as RNA positive for the indicated gene as described in Materials and Methods.

b The Fisher’s exact test was used to analyze the fraction of TG with detectable expression of viral lytic transcripts during latent infection in *n*212 versus *n*212R samples. TG with undetectable genomes were eliminated in this analysis.

c Data represent geometric means of numbers of RNA copies for the indicated genes per viral genome per TG.

d The Mann-Whitney test was used to analyze log (RNA copies/genome).

In summary, during latent infection of neurons, ICP0^+^ viruses showed increased heterochromatin association on the viral genome, particularly in the case of H3K27me3, and increased *LAT* and lytic transcript expression relative to ICP0^−^ viruses.

## DISCUSSION

HSV-1 ICP0 promotes viral lytic replication by promoting viral gene expression and inhibiting host restriction and innate immune mechanisms. The promotion of viral gene expression involves the reversal of the cellular epigenetic silencing of the viral genome through the degradation of the PML, Sp100, Daxx, ATRX, and IFI16 proteins, the disruption of the CoREST-HDAC complexes (8–14), and likely other mechanisms currently unknown. This results in increased transcription of all classes of lytic genes (36). Therefore, it has been believed that ICP0 expression would be counter to epigenetic mechanisms thought to silence gene expression during latent infection. Previous studies showed *ICP0* promoter activation in approximately one-third of latently infected neurons prior to the establishment of latency, VP16-independent *ICP0* promoter activation, and low levels of ICP0 expression during latency (39–41, 48). We also observed low but detectable levels of ICP0 expression during latency in at least some ganglia, in agreement with previous results. ICP0 has been known to promote establishment of and reactivation from latent infection (37, 38), but this was believed to be due to its effects on lytic infection during acute infection at peripheral sites or during reactivation. Although one publication had argued that ICP0 increased latent infection, that was in an *in vitro* neuronal infection model that included the use of the antiviral drug acyclovir to prevent viral replication in the cultures (43). However, cell culture models of quiescent HSV-1 infection found that ICP0 expression induced active lytic gene expression and enrichment of acetylated histones while preventing or reducing association of histone H3, H3K9me3, and HP1γ (49–51).

**FIG 6:**
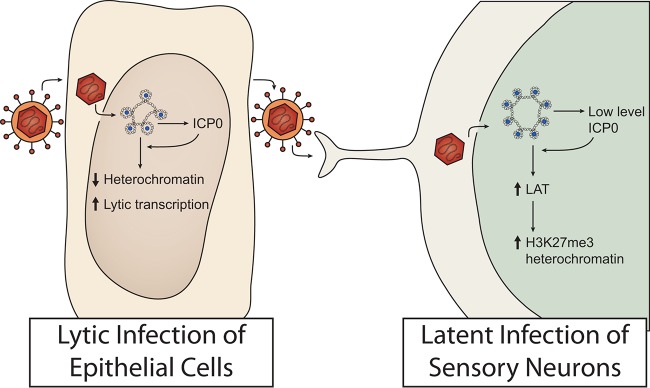
Model for epigenetic regulation of HSV-1 lytic and latent infection by the viral protein ICP0. In the nucleus of epithelial cells, HSV is targeted by host cell repression, which is countered by the viral ICP0 protein that functions to reduce histone occupancy and heterochromatin levels and promote lytic gene transcription. In contrast, during latent infection of neurons, the low levels of ICP0 expressed can promote the expression of *LAT* and the accumulation of H3K27me3 heterochromatin.

We were therefore surprised to observe that ICP0^−^ viruses showed reduced levels of H3K27me3 and H3K9me3 modified viral chromatin, which is generally considered to indicate the presence of heterochromatin. This phenotype was observed with two different types of *ICP0* gene mutants, a nonsense mutant and a promoter mutant. This argues that the phenotype is truly due to the absence of the ICP0 protein. We additionally found that an *ICP0* nonsense mutation resulted not only in a decrease in lytic gene expression but also, surprisingly, in a decrease in LAT expression. We therefore propose the following heuristic model (Fig. 6), which warrants further testing. In epithelial cells, ICP0 promotes the reversal of epigenetic silencing by allowing viral ICP4 to activate transcription of viral lytic promoters. In contrast, limited and/or intermittent expression of ICP0 in sensory neurons opens the chromatin sufficiently to increase transcription from not only the one highly active *LAT* promoter but also the weakly active lytic gene promoters. LAT then increases heterochromatin modifications, in particular, modification of H3K27me3. Each of these steps is discussed in depth below. It is also conceivable that ICP0 promotes the spread of HSV to different neuronal subtypes that load more chromatin and heterochromatin on the viral genome. However, in the absence of additional data supporting this, we favor the simpler model illustrated in Fig. 6.

### ICP0 and *LAT* transcription.

We propose that an early effect of ICP0 in sensory neurons is increased *LAT* transcription. ICP0 has the ability to promote transcription on transfected plasmids (52–55) and open chromatin on transfected plasmids (6) and to promote transcription (34, 36) and euchromatin formation (5) on the HSV genome in infected cells while having little effect on the cellular chromatin (56). Therefore, ICP0 may keep the chromatin open on all viral promoters, allowing resident transcription factors to function. It is well documented that in epithelial cells, ICP0 opens chromatin on lytic viral promoters, allowing activation by ICP4 and associated factors (2). In neurons, a region of open chromatin is maintained at the *LAT* promoter, which contains a variety of binding sites for transcription factors that are present in neurons (57). ICP0 may also at least transiently act on lytic promoters to induce an open or poised form in neurons so that the low level of relevant transcription factors can promote limited transcription of these genes. However, ICP0 induces only a small subset of cellular genes (56), and the basis for ICP0 specifically inducing expression of genes on plasmids and HSV genomes remains to be defined. Nonetheless, our results indicate that ICP0 can act on the HSV genome in sensory neurons during acute and/or latent infection to promote *LAT* and lytic gene transcription.

### ICP0 and heterochromatin in neurons.

We propose that the increase in heterochromatin levels in neurons as a result of ICP0 expression in ICP0^+^ virus-infected ganglia is an indirect effect of increased LAT transcription (Fig. 6). As shown previously, latent HSV-1 is associated with heterochromatin. However, we found that ICP0^+^ viral genomes are associated with increased levels of H3K27me3 and H3K9me3 heterochromatin at lytic gene promoters compared to those seen with ICP0^−^ viral genomes. The increased heterochromatin levels detected by ChIP were consistent with the increased proportion of nucleosome-bound DNA shown by FAIRE analysis, indicating a role for ICP0 in increased chromatin silencing during latent infection.

Interestingly, the significant increase in the level of H3K27me3 heterochromatin at the *LAT* promoter, *LAP*, did not appear to correlate with a reduction in *LAT* transcript accumulation by ICP0-expressing viruses. Similarly, it did not correlate with decreased expression of lytic transcripts. Instead, absolute levels of *LAT* were higher for the rescued viruses than for the mutant, suggesting that ICP0 may have a role in *LAT* regulation. *LAT* has previously been shown to increase H3K27me3 levels on viral lytic promoters (25). *LAP* has previously been shown to contain both repressive H3K27me3 and activating H3K4me3 modifications during latency, suggesting that H3K27me3 alone might not repress transcription at *LAT* (25). Bivalent cellular chromatin has been associated with poised transcription (58, 59), and bivalent modifications at the *LAT* promoter may be an important regulatory mechanism for *LAT* transcription. The apparent bivalency may also be due to distinct populations of genomes or distinct copies of *LAT* exhibiting variable chromatin association. *LAT* expression may be related to localization of viral genomes in proximity to cellular heterochromatin within neuronal cells (60). Advances in single-cell and single-molecule analysis of chromatin and gene expression should enable the resolution of these possibilities.

The increased heterochromatin levels associated with ICP0^+^ viruses could also indicate that ICP0 modulates chromatin in a cell type- or context-dependent manner. Our recent study identified specific demethylases and chromatin-modifying factors that operate on the *ICP8* promoter in a cell type-specific manner (61). Models for the mechanisms by which ICP0 disrupts silencing in epithelial cells during lytic infection include degradation of cellular IFI16, degradation of PML and disruption of PML bodies, and disruption of the repressive HDAC-CoREST-LSD1-REST complex by dissociating HDAC (12, 14, 62). These targets operate within complex pathways that may also exhibit cell type-specific activity, and novel mechanisms might additionally be operational in neurons. For instance, CoREST is known to function in a variety of complexes involved in tissue-specific silencing, such as the mSin3 complex which can be repressive or activating for dynamic promoter regulation, the repressive NRD complex, and a third HDAC1/2-containing complex lacking REST and RbAp46/48 components (63). Therefore, ICP0 may interact with chromatin-modifying complexes uniquely expressed or functionally specialized in neuronal cells to promote heterochromatin formation during latent infection. A screen for such host factors in neurons could provide additional information about the mechanisms of the apparent dual roles for ICP0 in the lytic-latent balance.

### Role for ICP0 in establishment and/or maintenance of latent infection.

The effect of ICP0 on latent infection could be exerted during establishment of latent infection or maintenance of latent infection, or both, through several mechanisms. The establishment of latent infection occurs gradually within neurons during and after the resolution of acute infection. Previous studies have shown that heterochromatin accumulates progressively as latent infection is established (15, 16). First, early expression of ICP0 may facilitate establishment of latent infection by increasing viral genome loads in the ganglia. Enhanced viral genome numbers, lytic gene expression, and/or ICP0 levels may provoke a cellular immune response leading to enhanced silencing. Low levels of lytic gene expression during latency have been correlated with changes in neuronal gene expression (18, 64, 65). This suggests that HSV-1 may actively modulate the cellular environment to facilitate latency. ICP0 may help maintain a low level of lytic gene expression, whereas viruses lacking ICP0 may be cleared after encountering a hostile neuronal environment refractory to the establishment of latency. Future characterization of chromatin-modifying complexes that interact with ICP0 in neuronal cells during establishment of latency could indicate how ICP0 functions to recruit silencing complexes in neurons.

Previous molecular studies have demonstrated that *ICP0* transcripts are expressed during latent infection (20, 39, 40) and that the *ICP0* promoter was active during establishment and/or maintenance of latent infection in a fraction of latently infected neurons (41). However, there has been little evidence that ICP0 protein is expressed or that it is functioning in neurons. Our analysis of an *ICP0* nonsense mutant virus provides strong genetic evidence that ICP0 protein is indeed expressed in neurons and has a function in promoting transcription and affecting latent viral chromatin.

### ICP0 as a target for antivirals affecting latent infection.

Our results argue for a role for ICP0 in promoting latent transcription and viral heterochromatin levels and raise the possibility that ICP0 might serve as a target for antivirals directed toward latent infection. A small-molecule inhibitor of ICP0 could reduce acute infection, establishment of latent infection, or reactivation from latent infection. Because all of the current HSV antivirals target other lytic infection targets such as the viral thymidine kinase and DNA polymerase, this report suggests an important new target for HSV drugs. In addition, small-molecule inhibitors could provide important research tools to probe the roles of ICP0 in establishment of latent infection versus maintenance. For example, administration of the inhibitors to mice during acute infection but after HSV has reached the sensory ganglia could test the role of ICP0 in establishment of latent infection. In contrast, administration of the inhibitors after latent infection has been established could test the role of ICP0 in maintenance of latent infection. The inhibitors could be tested in the mouse, rabbit, and guinea pig infection models as done with drugs affecting enzymes that modify chromatin (32).

Inasmuch as LAT expression and heterochromatin modifications promote latency, results from ICP0 studies represent, to our knowledge, the first genetic evidence that a herpesviral protein functions to promote both lytic and latent infection. A number of herpesviral lytic proteins are reported to be expressed during latent infection (1), but there is limited information about their functions. Human cytomegalovirus UL84 and UL44 proteins function during lytic infection and are reported to interact with the latent viral genome (66), but their latent function(s) has not been defined. Accordingly, the identification of small-molecule inhibitors that inhibit ICP0 may provide new latent infection therapeutics for HSV. The elucidation of other viral proteins that promote latent infection of other herpesviruses should greatly expand the kinds of therapeutics that we have for this medically important group of viruses.

## MATERIALS AND METHODS

### Cells and viruses.

U2OS (ATCC htb-96; ATCC, Manassas, VA) cells were maintained in Dulbecco’s modified essential medium (DMEM) supplemented with 5% (vol/vol) fetal calf serum, 5% (vol/vol) bovine calf serum, and 2 mM l-glutamine at 37°C. The HSV-1 *n*212 *ICP0* nonsense mutant virus, which contains a nonsense mutation in codon 212, has been described previously (44). The *n*212R ICP0^+^ rescued virus was constructed by homologous recombination of a full-length *ICP0*-containing plasmid with infectious *n*212 virus (6). The *d*Prom *ICP0*-promoter deletion mutant virus and the corresponding PromR ICP0^+^ rescued virus were constructed by homologous recombination with the *ICP0*-null 7134 virus (6). Stocks of ICP0^−^ and ICP0^+^ viruses were propagated and titrated in U2OS cells.

### Mouse infections and isolation of trigeminal ganglia.

Mice were housed in accordance with institutional and NIH guidelines on the care and use of animals in research, and all procedures were approved by the Institutional Animal Care and Use Committee of Harvard Medical School. Six-week-old CD1 male mice (Charles River Laboratories) were anesthetized in an isoflurane chamber followed by intraperitoneal injections of ketamine (3.7 mg/mouse) and xylazine hydrochloride (0.5 mg/mouse). The corneas were scarified, and infections were carried out at a dose of 2 × 10^4^ PFU/eye of ICP0^+^ viruses, PromR and *n*212R, and 2 × 10^6^ PFU/eye of ICP0^−^ viruses, *d*Prom and *n*212. These doses were chosen because they yielded equivalent levels of replication in the eye in the first 2 to 3 dpi. To measure infection in the eye, swabs of tear film were collected using sterile polyester applicators (Puritan) for the first 5 dpi, and virus in the tear films from the eye was titrated on U2OS cells as described previously (67). Survival was measured for 28 days, and the resulting data were analyzed using GraphPad Prism software. At 28 dpi, the animals were sacrificed and the latently infected trigeminal ganglia were dissected and immediately frozen in liquid nitrogen and stored at − 80°C.

### ChIP.

Chromatin immunoprecipitation (ChIP) analyses of TG were carried out as described previously (25) but with the following modifications. Intact TG were fixed at 37°C for 15 min in 20 ml of serum-free media containing 1% formaldehyde (16% formaldehyde ampules; Thermo Scientific). TG were then homogenized with Qiagen 5-mm-diameter stainless steel beads in a Qiagen TissueLyser LT 3 times at 50 oscillations per s for 2 min each time with a brief spin between steps. The homogenized samples were then sonicated at 4°C for about 9 cycles of 5 min each in a Diagenode Bioruptor on the high setting (15 s on, 45 s off) to shear the DNA to ~500-bp lengths. Immunoprecipitation reactions were carried out as described previously with 50 µg of chromatin and 2.5 µg of antibody (H3, Abcam ab1791; H3K9me3, Abcam ab8898; H3K27me3, Active Motif 39156; or negative-control rabbit IgG [Millipore NG1893918]) per IP reaction mixture incubated at 4°C overnight. Immunocomplexes were isolated with MagnaChIP magnetic beads and washed 3 times with cold low-salt buffer (150 mM NaCl, 20 mM Tris-HCl [pH 8.1], 2 mM EDTA, 1% Triton X-100, 0.1% SDS, 1 mM phenylmethylsulfonyl fluoride [PMSF]), 3 times with cold LiCl wash buffer (50 mM HEPES [pH 7.5], 250 mM LiCl, 1 mM EDTA, 1% NP-40, 0.7% sodium deoxycholate, 1 mM PMSF), and once with cold Tris-EDTA buffer (10 mM Tris-HCl [pH 8], 1 mM EDTA). The chromatin complexes were eluted by incubation of the beads in 90 µl of elution buffer (1% SDS, 0.1 M NaHCO_3_) at 65°C for 10 min followed by rotation for 10 min at room temperature performed twice for a 180-µl total elution volume. DNA was then isolated by reversal of formaldehyde cross-links and incubation with RNase A followed by proteinase K and purification with a QIAquick Qiagen PCR purification kit and was eluted twice to yield a 100-µl total volume.

### FAIRE.

Formaldehyde-assisted isolation of regulatory elements (FAIRE) was carried out on latently infected ganglia using a previously described protocol (47). TG were fixed, homogenized, and sonicated as described above for the ChIP experiments. For FAIRE analysis, 15 µg of total chromatin was diluted to a total volume of 200 µl in ChIP dilution buffer (150 mM NaCl, 10 mM Na_2_HPO_4_, 2 mM EDTA, 1.1% Triton, 0.1% SDS). Without reversing DNA-histone cross-links, nucleosome-free DNA was extracted using an equal volume of phenol-chloroform-isoamyl alcohol, followed by ethanol precipitation. DNA-histone cross-links were reversed in inputs processed in parallel containing equal amounts of chromatin, and then phenol-chloroform was extracted. The nucleosome-free DNA was then quantified by real-time PCR and expressed relative to the input DNA isolated.

### Real-time PCR for DNA quantification of ChIP.

Quantitative real-time PCR was performed using Power SYBR green PCR master mix and a Prism 7300 real-time system (Applied Biosystems) according to the manufacturer’s instructions. The final reaction volume was 25 µl and contained 2.5 µl DNA and a 100 nM concentration of each primer. The specificity of each primer pair was determined by running a dissociation curve analysis of the PCR products for each reaction. All DNA samples were run in duplicate, and relative copy numbers were determined by comparison with a standard curve generated using 10-fold serial dilutions of HSV-infected cell DNA. The fraction of viral DNA immunoprecipitated relative to the 10% input was normalized to the fraction of cellular GAPDH (glyceraldehyde 3-phosphate dehydrogenase) DNA immunoprecipitated relative to 10% input in the same reaction. The primers used (16, 25) are listed in Table 2.

**TABLE 2:** Sequences of primers

Primer	Sequence
ICP8 F	GAGACCGGGGTTGGGGAATGAATC
ICP8 R	CCCCGGGGGTTGTCTGTGAAGG
ICP27 F	ACCCAGCCAGCGTATCCACC
ICP27 R	ACACCATAAGTACGTGGCATGT
LAP F	CCCGGCCCGCACGAT
LAP R	CAACACCCCGCCGCTTT
LAT 5′ exon F	TTCGTTTTCCCGTTTCG
LAT 5′ exon R	CAGACGGGTTAAAGAAACAGAAACC
GAPDH F	CAGGCGCCCAATACGACCAAAATC
GAPDH R	TTCGACAGTCAGTCAGCCGCATCTTCTT

### DNA and RNA isolation and quantification of viral transcripts.

Trigeminal ganglia were isolated from HSV-1-infected mice and immediately frozen in liquid nitrogen and stored at −80°C. DNA and RNA were isolated using an Allprep DNA/RNA minikit (Qiagen) following the manufacturer’s instructions. Reverse transcription was performed using a QuantiTect Rt. kit (Qiagen) following the manufacturer’s instructions, except using specific primers as described previously (33). Viral DNA and transcripts were quantified relative to host DNA and a control mRNA using serially diluted standards as described previously (15, 25, 33, 68). Standard curves were linear in all cases (with *R*^2^ values over 0.99). The detection limit of the ICP0, ICP27, tk, and gC assays was 100 copies/TG. In addition, for the data presented in Table 1, the presence of transcripts scored as detectable was confirmed by the presence of transcript-specific peaks in qPCR melt curves that were absent in all the mock-infected samples tested. The fraction of lytic RNA-positive ganglia in mutant and rescued virus infections was statistically analyzed by Fisher’s exact test, and differences in levels of transcripts per genome were scored using a Mann-Whitney test. For quantitation of transcripts, undetectable values were set at 1 transcript/ganglion.
